# Dental Caries—What It Means to the Work of the Physician

**Published:** 1917-01

**Authors:** C. M. M’Cauley

**Affiliations:** Dallas, Texas


### Dental Caries—What it Means to the Work of the Physician.

BY C. M. M'CAULEY, D. D. S., DALLAS, TEXAS.

"Prevention" is the great slogan of the twentieth century physician. He has endeared himself to the hearts of the people by his unselfish efforts to ascertain the cause and learn to prevent the various diseases which are shortening the lives of many and filling others with pain and discomfort. But for the ravages of preventable diseases the majority of lives would be longer, happier and more useful to the world. There would be less crime and less poverty. There would be more of the elements of exalted life and the world would be better generally. In view of these things, is it any wonder that the true, unselfish physician is loved by all?

After the stage of "prevention" has passed, the first thought of the modern physician is to "remove the cause." Every physician who is laboring in the work of prevention should be vitally interested in the prevention of dental caries; likewise every physician who seeks the cause of illness before trying to cure it should never overlook dental caries. I do not mean to say that in the mouth can be found the cause of all ills, but I do say that sufficient ills have their origin in the mouth to warrant the thorough physician in always taking that source of illness into consideration when seeking the cause of any infectious disease.

Caries of teeth is itself a disease which is preventable, and the dental profession has so recognized it for a generation past, during which time their efforts have been directed toward teaching the people the importance of prevention. During the last five years many millions of dollars have been spent to impress this fact upon the people, and to assist the poor to eradicate the disease.

Dental caries is the most prevalent disease known to man. It is derived from two sources or causes, namely: first, unsanitary condition of mouths; second, defects of development.

Such an overwhelming percentage of caries is caused by neglect that we shall consider that practically the only cause. Food debris accumulates between the teeth and over the surfaces and a placque is formed on the enamel surface. Micro-organisms lodge in this placque and secrete an acid which disintegrates the enamel. This is the beginning of caries. Unless the placque is allowed to form on the enamel surface the micro-organisms cannot lodge there and secrete the acid. If a brush is passed well over that surface every twenty-four hours the placque cannot form; hence it follows that upon surfaces cleansed by a brush, or otherwise, as often as once in twenty-four hours there will be no decay of tooth structure. Where the difficulty comes is in brushing and cleansing all the tooth surfaces. The whole secret of prevention of the first classification of caries lies in knowing how to cleanse every part of the teeth and putting that knowledge into daily practice.

The second classification which has its origin in developmental defects which constitutes a very small percentage of existing caries, and which can be prevented by repairing the defects before caries have made its inroads upon the tooth structure to any perceptible extent. Some of the causes of these defects are rickets, scarlet fever during the early years of life while the teeth are calcifying, insufficient nourishment during fetal life, etc. The bottle-fed baby fails to get the thyroid element in the mother's milk, and this is thought to have a deleterious influence upon tooth formation. Most of such defects as these can be easily corrected during childhood if the advice and services of a competent dentist are secured.

Some of the effects of dental caries upon the general health are as follows:

The unclean condition in the mouth causes most caries, also causes stomach disturbance by the ingestion of unclean, unsanitary food. The mouth is one of the best breeding places for germ life, and when unclean its properties, as an incubator, are wonderfully increased. The cavities in the teeth add materially to its breeding and carrying facilities. As a carrier of disease, the unclean mouth containing carious teeth ranks very high. The owner of this "cesspool" mouth uses it upon drinking cups at public fountains, drug stores, restaurant and hotel dishes; uses it to expectorate these masses of germ life of various kinds upon the streets and in many public places. By one means or another many of these deadly germs find their way into the bodies of others where a fertile soil is found and illness and death are the result. Not only does the cavity in the tooth facilitate this dreadful work, but the condition which produces the cavity will do almost as much. During the progress of decay the tooth usually becomes sensitive and lame. Mastication is interfered with and the stomach suffers overwork and perhaps irritation. When decay reaches the nerve or pulp cavity it brings the germ life into contact with the nervous and the circulatory systems. Following this is possible intense pain which may disturb the whole body and possibly lower the opsonic index temporarily. There is also a possibility that infection may enter the circulation from the debris in the tooth cavity. A little later or in this same case the pulp tissue dies and disintegrates.

Following this disintegration which is associated with gas formation an abscess may form at the apex of the root. Much suffering and general systemic disturbance is often experienced when the abscess first forms and until it opens and allows the escape of pus. If unattended this abscess remains there indefinitely, painlessly, but constantly forming and giving off pus, which may either find its way directly into the circulation, or may enter by way of the alimentary tract. There are millions of just such abscesses in daily operation in this country. The evils which they may do as a focus of infection is known to no one better than to your medical profession. There are also blind abscesses which came originally from dental caries,—and which are to be located only by the aid of the X-ray—found in mouths of those suffering from such ills as arthritis, nephritis, pericarditis, etc.

What is the remedy for all of this trouble? It is simply this, continue the campaign which is already started—teaching the people the means of prevention. The simple rules of cleanliness of the mouth. In this work we could have no better helpers than the physician. If the physician would give as much attention to the condition of the mouth of his hospital patient as he does to the purity of the food given the patient it seems reasonable that the patient would improve more rapidly, for it is certain that when the very best food is passed through a sewer on its way to the stomach it will become polluted by the contents of the sewer.

The children in the public schools must be taught the importance of a clean mouth. Not only that but they must be required to keep their mouths clean. A child should no more be permitted to attend school with an unclean, germ-laden mouth than he should be if he had measles or scarlet fever. He is a carrier of disease in both cases.

The physician is the co-worker of the dentist in the prevention and relief of suffering. There never was a time when this co-operation could be brought into active practice to better advantage than now. Dentistry needs the assistance of the medical profession more than that of any other people or organization in putting into effect their practical principles of prevention. Every city should have its public dispensary to care for the dental troubles of the poor. Not over 10 per cent of the whole people of this country are receiving dental treatment. Every public and private school should have the services of a competent teacher of mouth hygiene. The campaign of prevention on the part of the medical profession could be wonderfully augmented and facilitated by fostering this campaign of ours. Our profession needs better educational facilities in this State; you can help us secure them. We need legislation; you can help us with your strong organization. We need a word of advice from the family physician to his patient; you can do that. Dr. Mayo said the next great step in preventive medicine was to be taken by the dental profession. We are getting ready to make that step. Will you by your co-operation help us to reach the goal much sooner than we could without your help?

